# Supplementary material for: A multi-ancestry GWAS of Fuchs corneal dystrophy highlights the contributions of laminins, collagen, and endothelial cell regulation
Source: Commun Biol. 2024 Apr 6;7:418. doi: 10.1038/s42003-024-06046-3 (PMC10998918; doi:10.1038/s42003-024-06046-3)
Supplement: Supplementary file 2 — Supplementary Information [file 42003_2024_6046_MOESM2_ESM.pdf]

Supplementary information

A multi-ancestry GWAS of Fuchs corneal dystrophy highlights the contributions of laminins, collagen, and endothelial cell regulation

Bryan R. Gorman[1,2,28], Michael Francis[1,2,28], Cari L. Nealon[3], Christopher W. Halladay[4], Nalvi Duro[1,2], Kyriacos Markianos[1], Giulio Genovese[5,6,7], Pirro Hysi[8,9,10], Hélène Choquet[11], Natalie A. Afshari[12], Yi-Ju Li[13], VA Million Veteran Program*, J. Michael Gaziano[14,15], Adriana M. Hung[16,17,18], Wen-Chih Wu[19], Paul B. Greenberg[20,21], Saiju Pyarajan[1], Jonathan H. Lass[22], Neal S. Peachey[23,24,25,29], Sudha K. Iyengar[23,26,27,29]

[1]Center for Data and Computational Sciences (C-DACS), VA Boston Healthcare System, Boston, MA, USA; [2]Booz Allen Hamilton, McLean, VA, USA; [3]Eye Clinic, VA Northeast Ohio Healthcare System, Cleveland, OH, USA; [4]Center of Innovation in Long Term Services and Supports, Providence VA Medical Center, Providence, RI, USA; [5]Program in Medical and Population Genetics, Broad Institute of MIT and Harvard, Cambridge, MA, USA; [6]Stanley Center, Broad Institute of MIT and Harvard, Cambridge, MA, USA; [7]Department of Genetics, Harvard Medical School, Boston, MA, USA; [8]Department of Ophthalmology, King's College London, London, UK; [9]Department of Twins Research and Genetic Epidemiology, King's College London, London, UK; [10]UCL Great Ormond Street Hospital Institute of Child Health, King's College London, London, UK; [11]Division of Research, Kaiser Permanente Northern California (KPNC), Oakland, CA, USA; [12]Shiley Eye Institute, Viterbi Family Department of Ophthalmology, University of California, San Diego, La Jolla, CA, USA; [13]Department of Biostatistics and Bioinformatics, Duke University School of Medicine, Durham, NC, USA; [14]Massachusetts Veterans Epidemiology Research and Information Center (MAVERIC), VA Boston Healthcare System, Boston, MA, USA; [15]Division of Aging, Department of Medicine, Brigham and Women's Hospital, Harvard Medical School, Boston, MA, USA; [16]Division of Nephrology and Hypertension, Department of Medicine, Vanderbilt University Medical Center, Nashville, TN, USA; [17]Vanderbilt Center for Kidney Disease, Vanderbilt University Medical Center, Nashville, TN, USA; [18]VA Tennessee Valley Healthcare System, Nashville, TN, USA; [19]Cardiology Section, Medical Service, Providence VA Medical Center, Providence, RI, USA; [20]Ophthalmology Section, Providence VA Medical Center, Providence, RI, USA; [21]Division of Ophthalmology, Alpert Medical School, Brown University, Providence, RI, USA; [22]Department of Ophthalmology and Visual Sciences, Case Western Reserve University, Cleveland, OH, USA; [23]Research Service, VA Northeast Ohio Healthcare System, Cleveland, OH, USA; [24]Cole Eye Institute, Cleveland Clinic Foundation, Cleveland, OH, USA; [25]Department of Ophthalmology, Cleveland Clinic Lerner College of Medicine of Case Western Reserve University, Cleveland, OH, USA; [26]Cleveland Institute for Computational Biology, Case Western Reserve University, Cleveland, OH, USA; [27]Department of Population and Quantitative Health Sciences, Case Western Reserve University School of Medicine, Cleveland, OH, USA

*Lists of authors and their affiliations appear at the end of the paper.
[28]These authors contributed equally to this work.
[29]These authors jointly supervised this work.

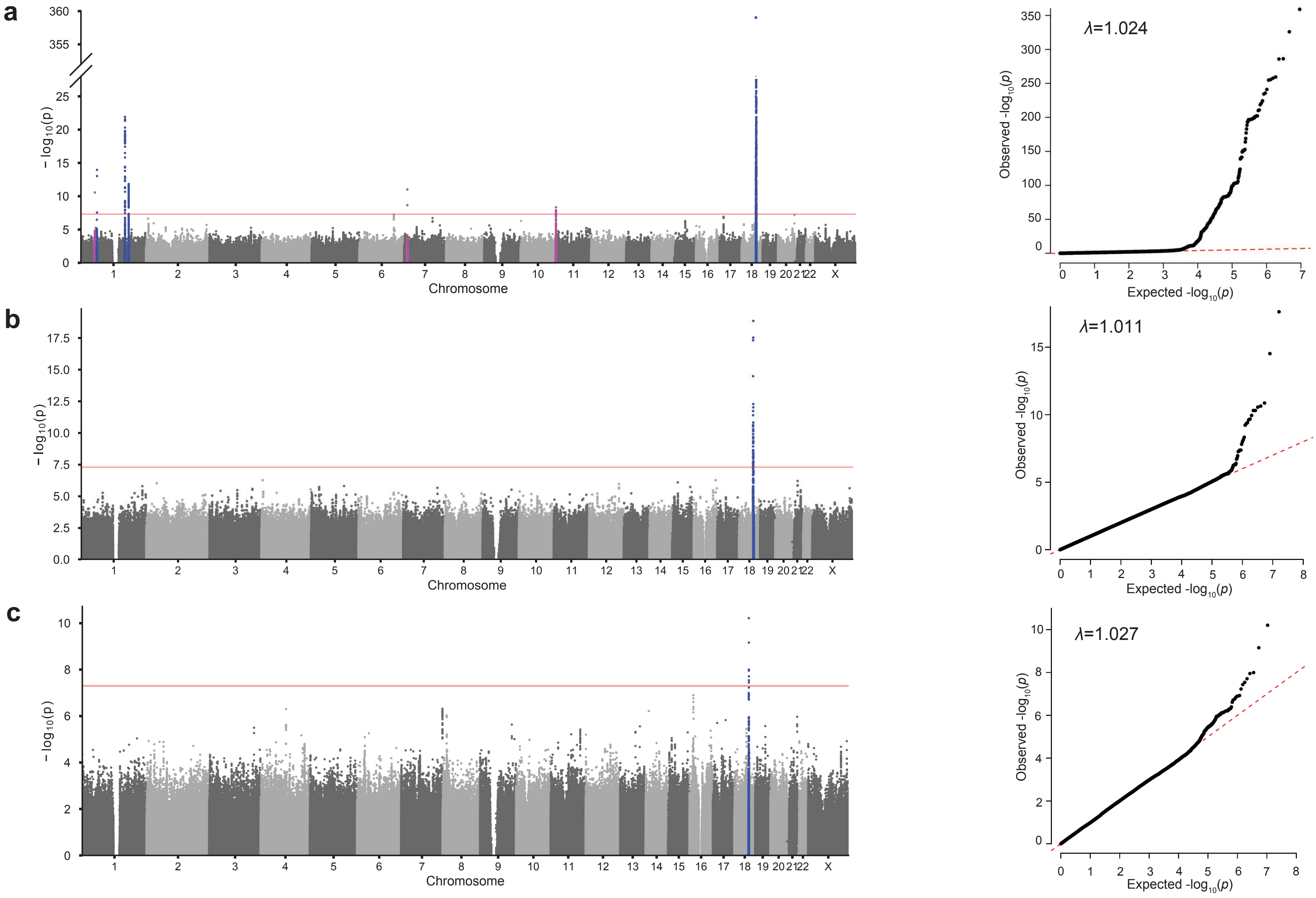

Supplementary Fig. 1. Manhattan and quantile-quantile (QQ) plots. Left: Manhattan plots showing -log$_{10}$(*p*) for associations of genetic variants with Fuchs endothelial corneal dystrophy in (**a**) European, (**b**) admixed African, and (**c**) Hispanic/Latino Million Veteran Program (MVP) cohorts. The red line indicates the genome-wide significance threshold ($P<5\times10^{-8}$). Novel loci are highlighted in red while known loci are highlighted in blue. Y-axis breaks in (**a**) are used to include the most significant variant at *TCF4*. **Right:** QQ plots showing observed versus expected distributions of association *P*-values.

a

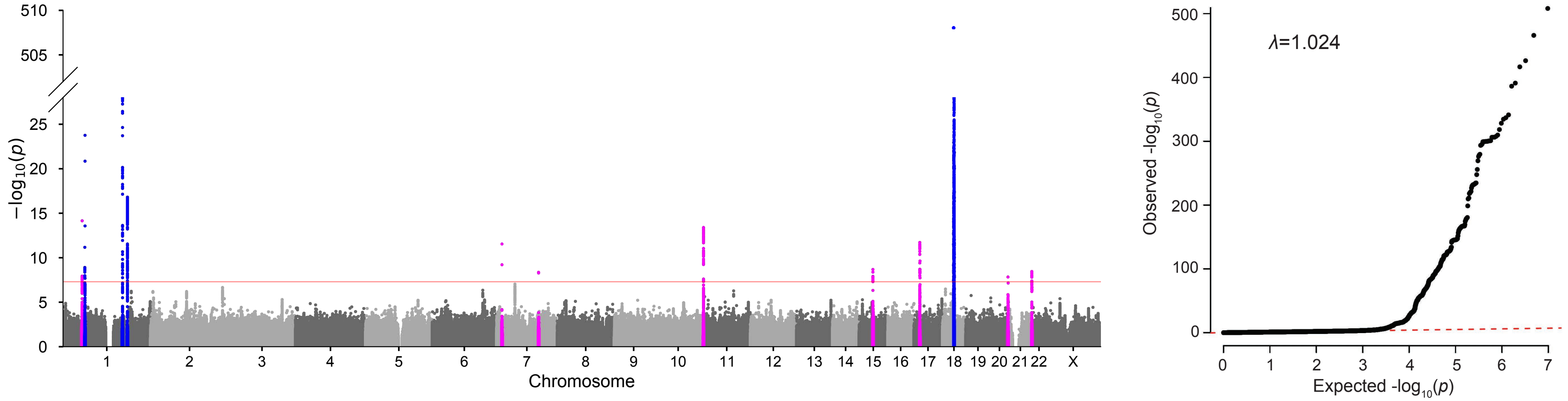

b

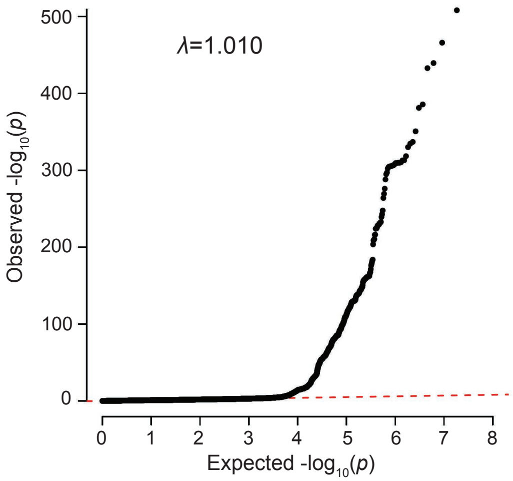

Supplementary Fig. 2. Meta-analysis Manhattan and quantile-quantile (QQ) plots. (**a**) Manhattan and QQ plot for European-only meta-analysis of MVP European and Afshari et al.[1] cohorts. The red line indicates the genome-wide significance threshold ($P<5\times10^{-8}$). Novel loci are highlighted in red while known loci are highlighted in blue. A Y-axis break is used to include the most significant variant at *TCF4*. (**b**) QQ plot for multi-ancestry meta-analysis (corresponding Manhattan plot shown in Main Figure 2).

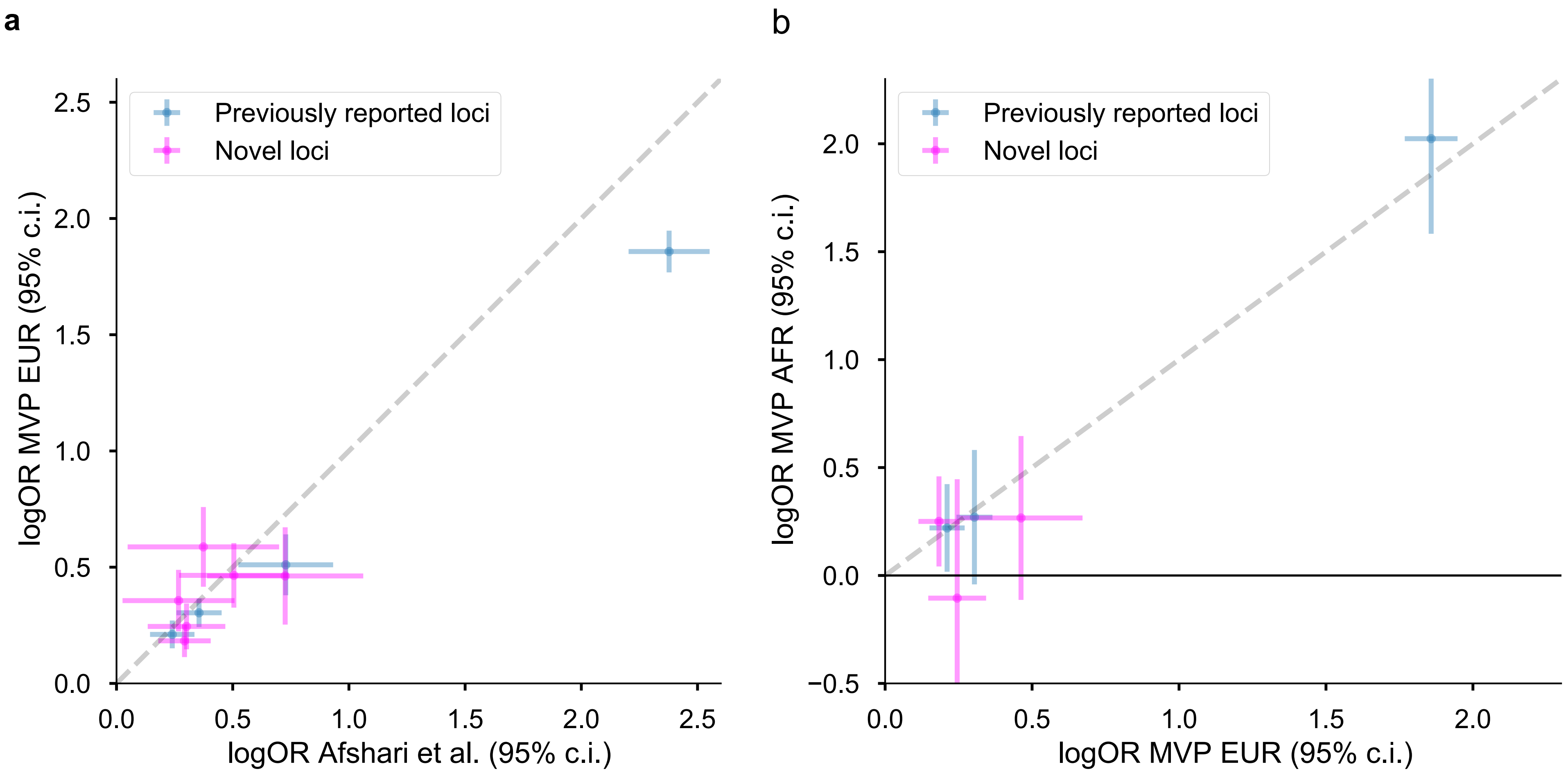

Supplementary Fig. 3. Comparison of allele effect sizes. Scatterplots comparing effect sizes (logarithm of odds ratios and 95% confidence interval) of previously reported (blue) and novel (pink) lead SNPs between **(a)** Million Veteran Program (MVP) European (EUR) and Afshari et al.[1], and **(b)** MVP admixed African (AFR) vs MVP EUR. Directions of effects estimates between cohorts were consistent for all lead SNPs except one, rs12439253 (*RORA*), which was not significant in AFR.

a

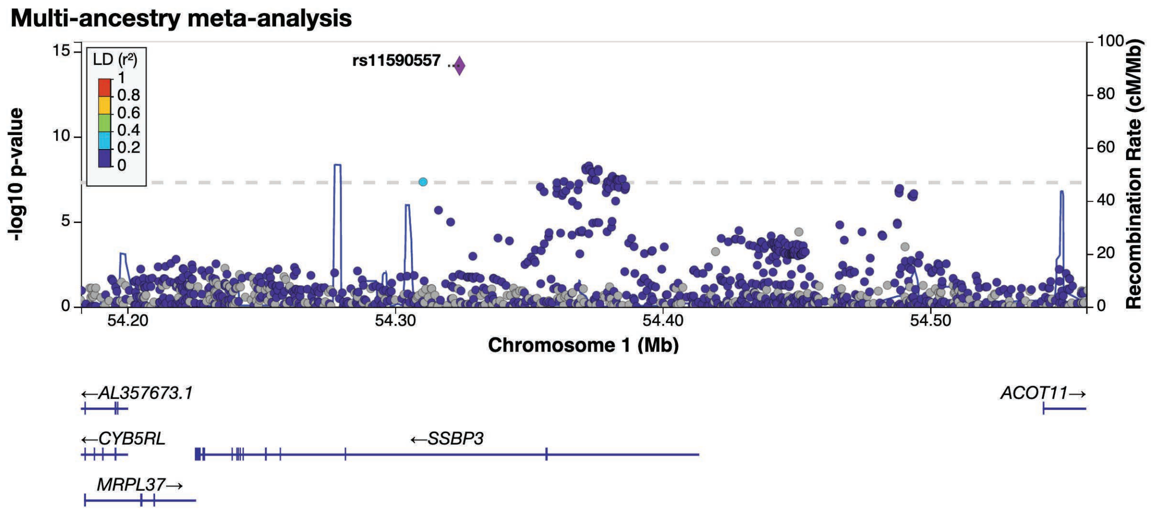

b

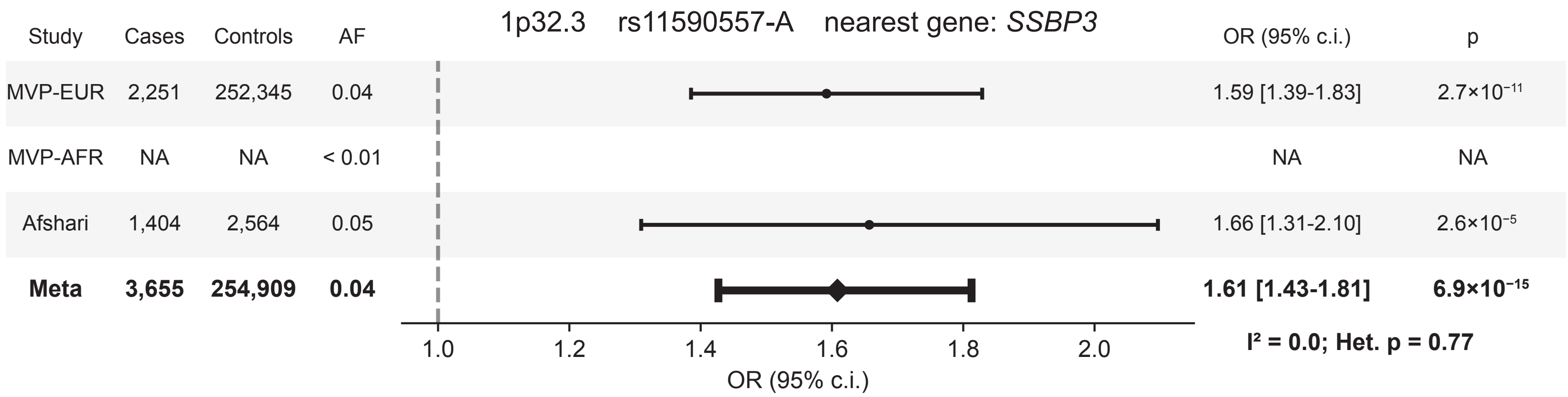

Supplementary Fig. 4. Regional Manhattan plot and forest plot for *SSBP3*. (a) LocusZoom regional Manhattan plot showing variant-level associations and local linkage disequilibrium (LD) structure of a significant multi-ancestry meta-analysis FECD genomic risk locus. Plots are presented in the order of their genomic coordinates. A European LD reference panel (1000 genomes) was used to color points in all plots. **(b)** Odds ratios (OR) and 95% confidence intervals for FECD at lead SNPs in Million Veteran Program European (EUR), admixed African (AFR), and Afshari[1] cohorts, and combined multi-ancestry meta-analysis (Meta). OR is not shown for six of twelve AFR index variants which did not meet the meta-analysis minor allele frequency cutoff of ≥1%; for these variants, “Meta” refers to the EUR-only meta-analysis.

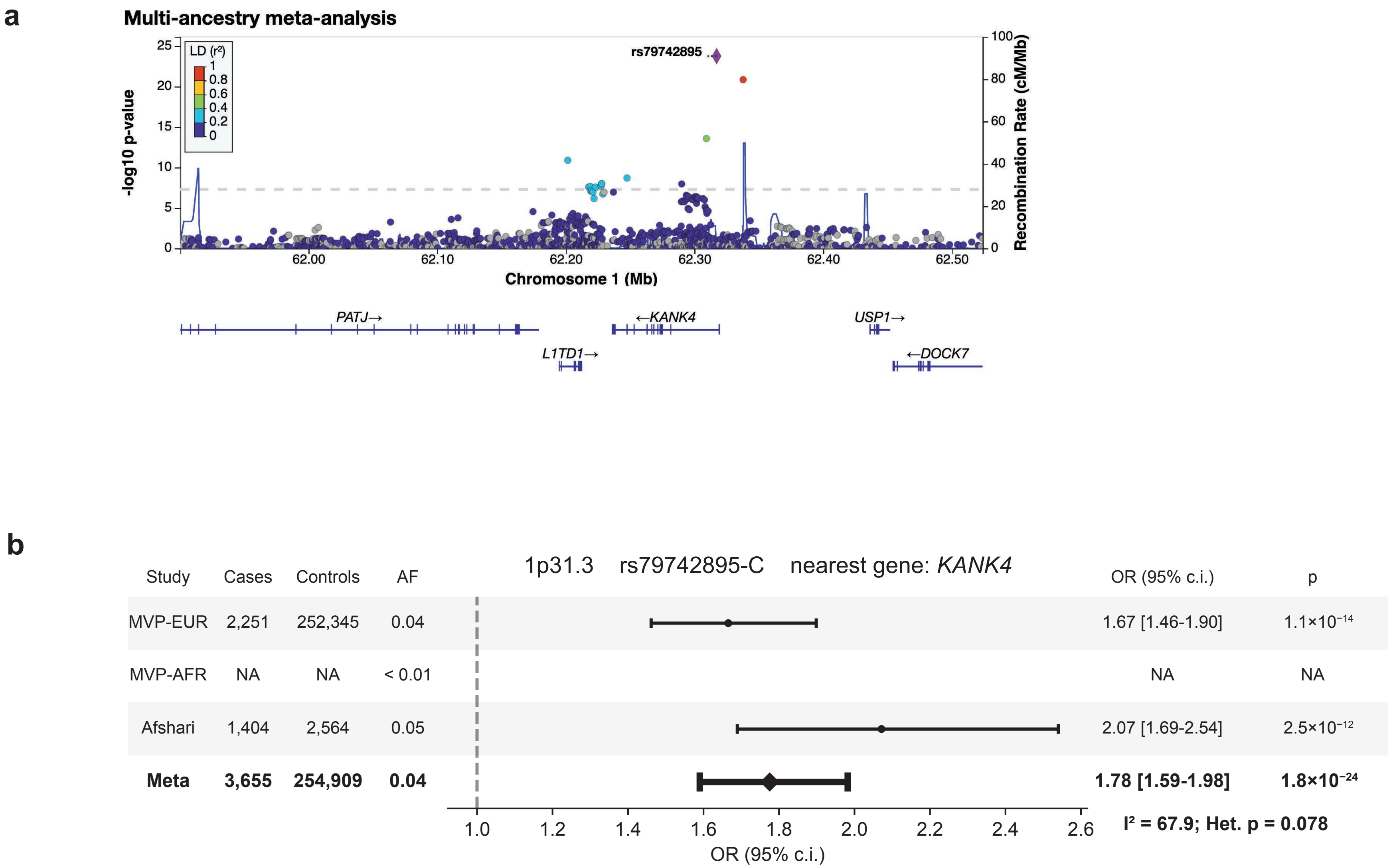

Supplementary Fig. 5. Regional Manhattan plot and forest plot for *KANK4*. (a) LocusZoom regional Manhattan plot showing variant-level associations and local linkage disequilibrium (LD) structure of a significant multi-ancestry meta-analysis FECD genomic risk locus. Plots are presented in the order of their genomic coordinates. A European LD reference panel (1000 genomes) was used to color points in all plots. **(b)** Odds ratios (OR) and 95% confidence intervals for FECD at lead SNPs in Million Veteran Program European (EUR), admixed African (AFR), and Afshari[1] cohorts, and combined multi-ancestry meta-analysis (Meta). OR is not shown for six of twelve AFR index variants which did not meet the meta-analysis minor allele frequency cutoff of ≥1%; for these variants, "Meta" refers to the EUR-only meta-analysis.

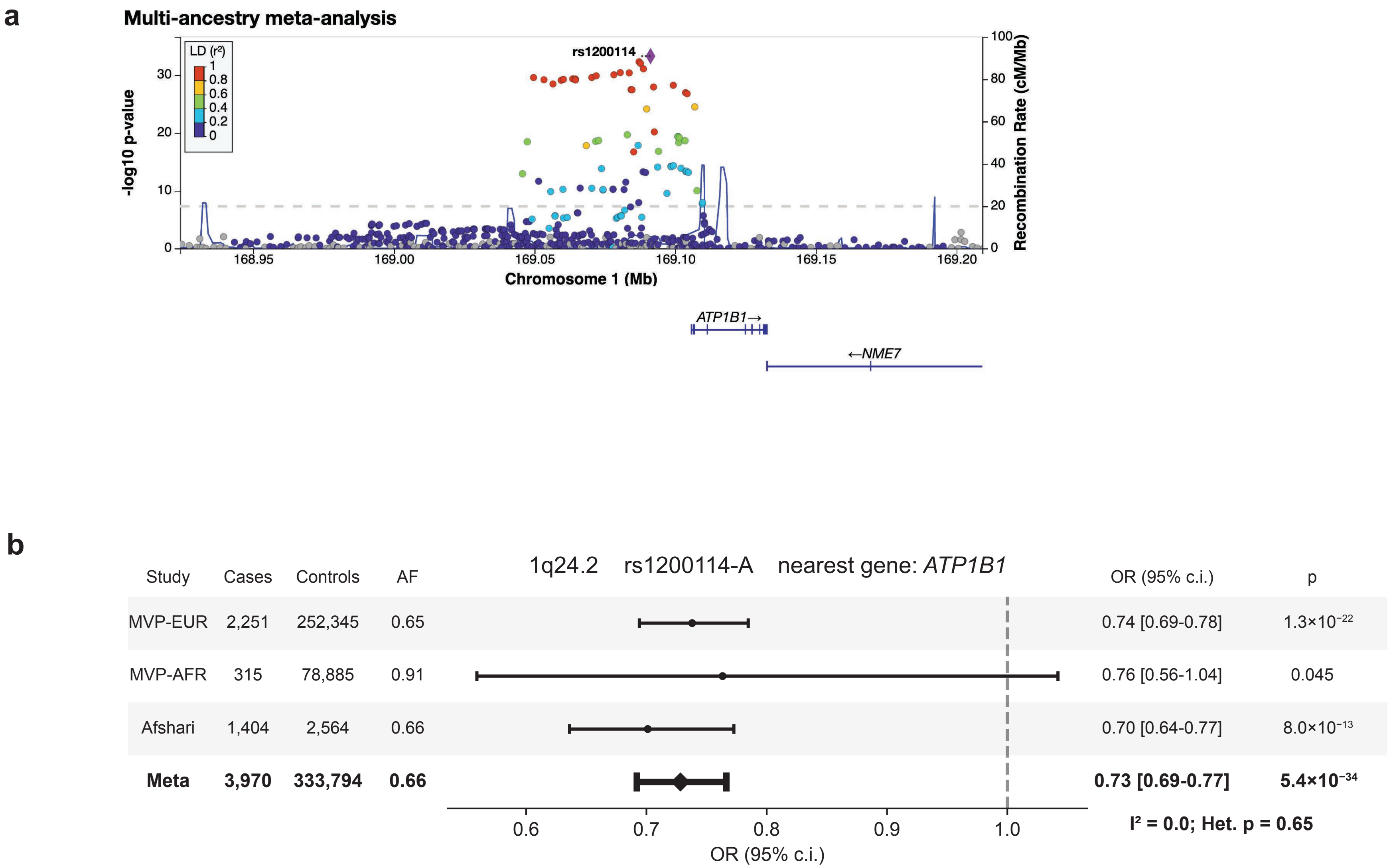

Supplementary Fig. 6. Regional Manhattan plot and forest plot for *ATP1B1*. (a) LocusZoom regional Manhattan plot showing variant-level associations and local linkage disequilibrium (LD) structure of a significant multi-ancestry meta-analysis FECD genomic risk locus. Plots are presented in the order of their genomic coordinates. A European LD reference panel (1000 genomes) was used to color points in all plots. **(b)** Odds ratios (OR) and 95% confidence intervals for FECD at lead SNPs in Million Veteran Program European (EUR), admixed African (AFR), and Afshari[1] cohorts, and combined multi-ancestry meta-analysis (Meta). OR is not shown for six of twelve AFR index variants which did not meet the meta-analysis minor allele frequency cutoff of ≥1%; for these variants, "Meta" refers to the EUR-only meta-analysis.

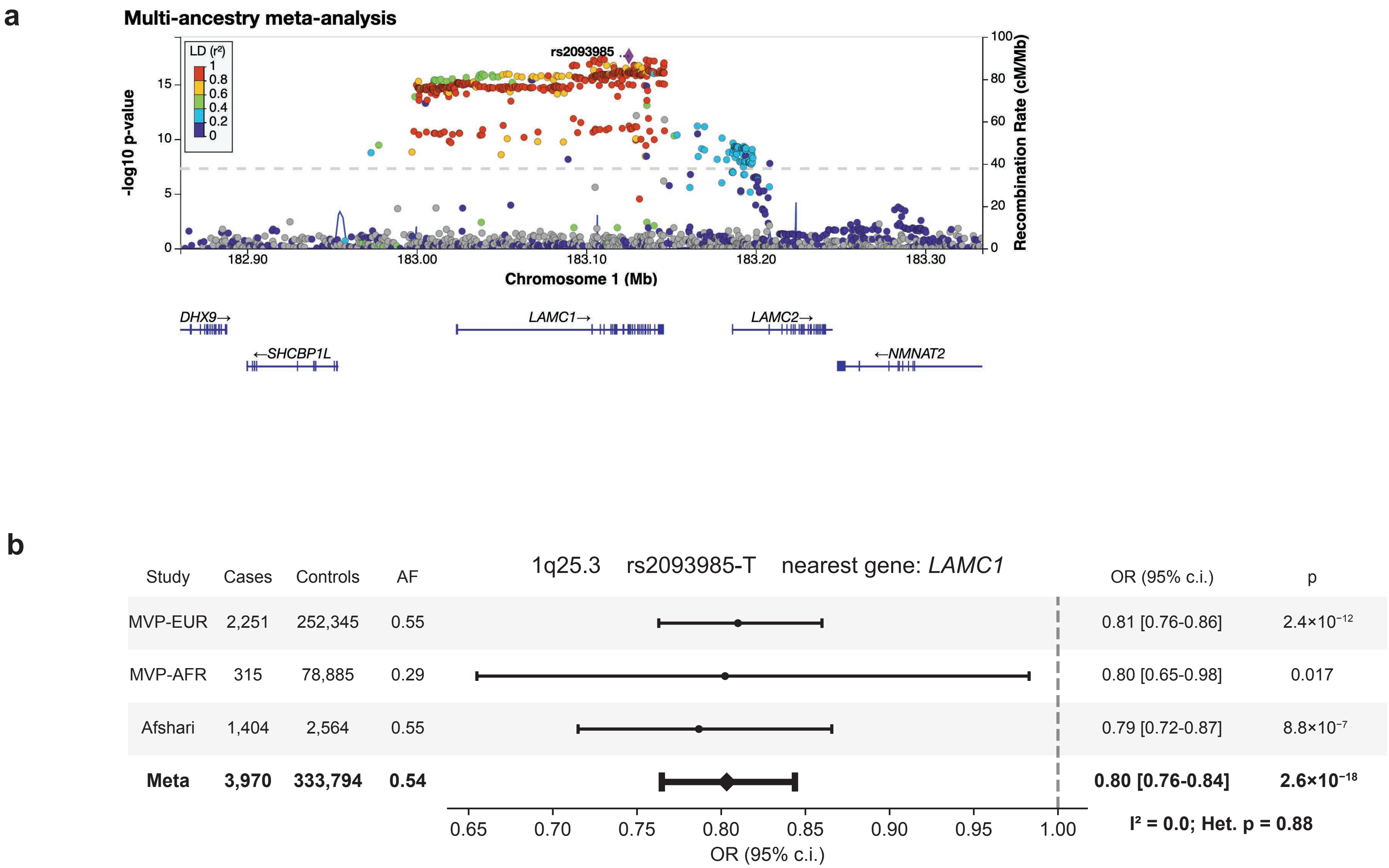

Supplementary Fig. 7. Regional Manhattan plot and forest plot for *LAMC1*. (a) LocusZoom regional Manhattan plot showing variant-level associations and local linkage disequilibrium (LD) structure of a significant multi-ancestry meta-analysis FECD genomic risk locus. Plots are presented in the order of their genomic coordinates. A European LD reference panel (1000 genomes) was used to color points in all plots. **(b)** Odds ratios (OR) and 95% confidence intervals for FECD at lead SNPs in Million Veteran Program European (EUR), admixed African (AFR), and Afshari[1] cohorts, and combined multi-ancestry meta-analysis (Meta). OR is not shown for six of twelve AFR index variants which did not meet the meta-analysis minor allele frequency cutoff of ≥1%; for these variants, "Meta" refers to the EUR-only meta-analysis.

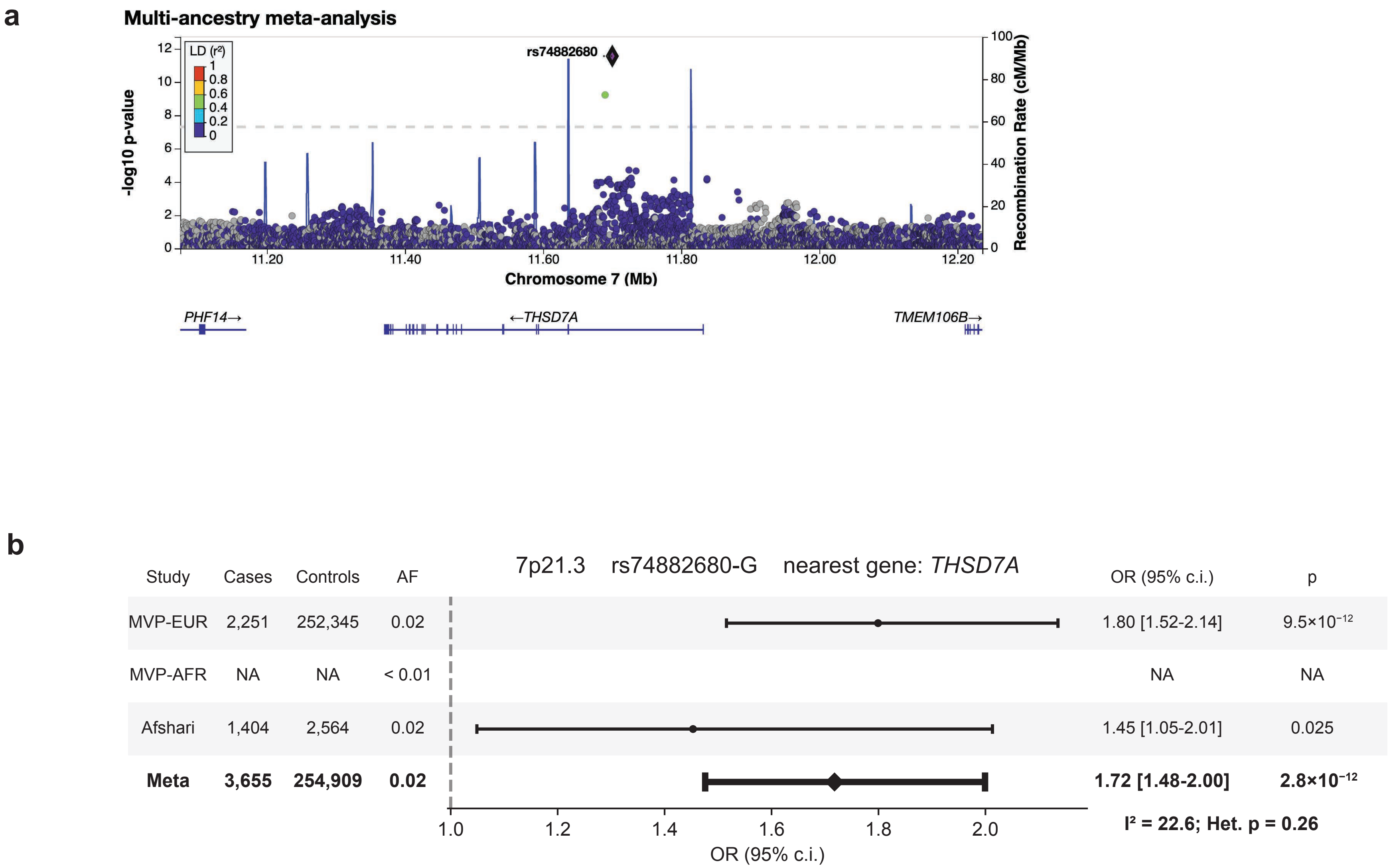

Supplementary Fig. 8. Regional Manhattan plot and forest plot for *THSD7A*. (a) LocusZoom regional Manhattan plot showing variant-level associations and local linkage disequilibrium (LD) structure of a significant multi-ancestry meta-analysis FECD genomic risk locus. Plots are presented in the order of their genomic coordinates. A European LD reference panel (1000 genomes) was used to color points in all plots. **(b)** Odds ratios (OR) and 95% confidence intervals for FECD at lead SNPs in Million Veteran Program European (EUR), admixed African (AFR), and Afshari[1] cohorts, and combined multi-ancestry meta-analysis (Meta). OR is not shown for six of twelve AFR index variants which did not meet the meta-analysis minor allele frequency cutoff of ≥1%; for these variants, "Meta" refers to the EUR-only meta-analysis.

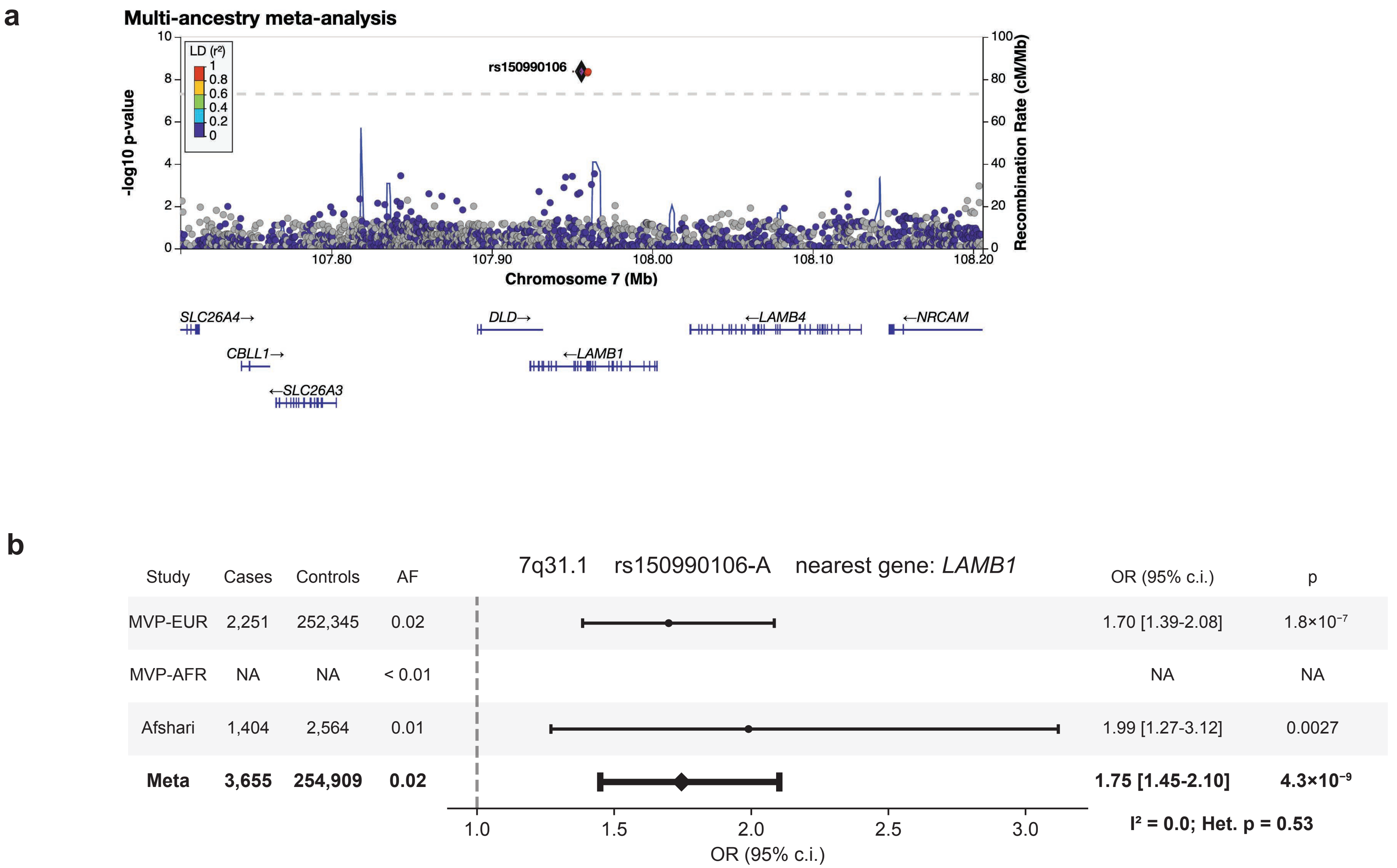

Supplementary Fig. 9. Regional Manhattan plot and forest plot for *LAMB1*. (a) LocusZoom regional Manhattan plot showing variant-level associations and local linkage disequilibrium (LD) structure of a significant multi-ancestry meta-analysis FECD genomic risk locus. Plots are presented in the order of their genomic coordinates. A European LD reference panel (1000 genomes) was used to color points in all plots. **(b)** Odds ratios (OR) and 95% confidence intervals for FECD at lead SNPs in Million Veteran Program European (EUR), admixed African (AFR), and Afshari[1] cohorts, and combined multi-ancestry meta-analysis (Meta). OR is not shown for six of twelve AFR index variants which did not meet the meta-analysis minor allele frequency cutoff of ≥1%; for these variants, "Meta" refers to the EUR-only meta-analysis.

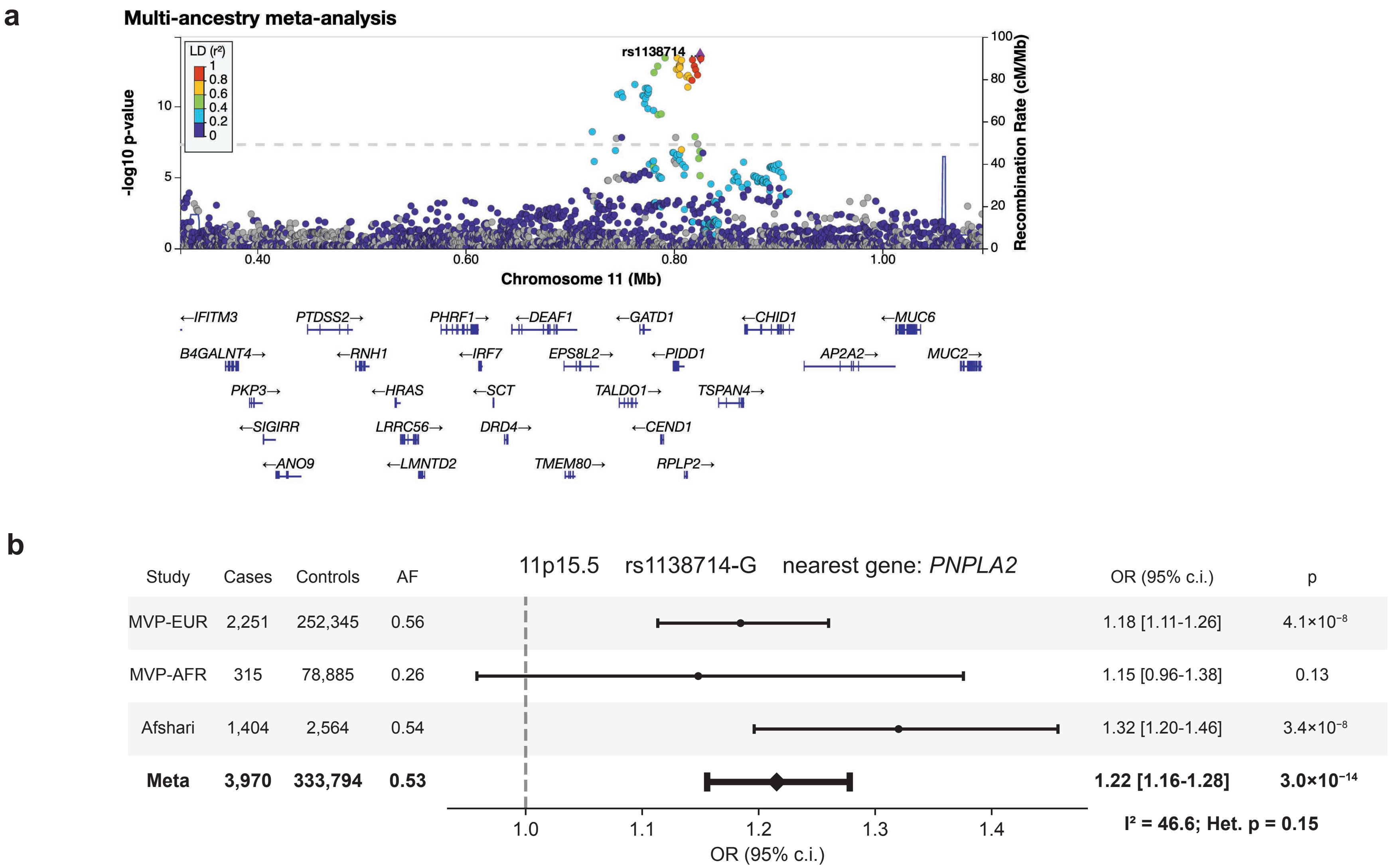

Supplementary Fig. 10. Regional Manhattan plot and forest plot for *PNPLA2/PIDD1*. (a) LocusZoom regional Manhattan plot showing variant-level associations and local linkage disequilibrium (LD) structure of a significant multi-ancestry meta-analysis FECD genomic risk locus. Plots are presented in the order of their genomic coordinates. A European LD reference panel (1000 genomes) was used to color points in all plots. **(b)** Odds ratios (OR) and 95% confidence intervals for FECD at lead SNPs in Million Veteran Program European (EUR), admixed African (AFR), and Afshari[1] cohorts, and combined multi-ancestry meta-analysis (Meta). OR is not shown for six of twelve AFR index variants which did not meet the meta-analysis minor allele frequency cutoff of ≥1%; for these variants, "Meta" refers to the EUR-only meta-analysis.

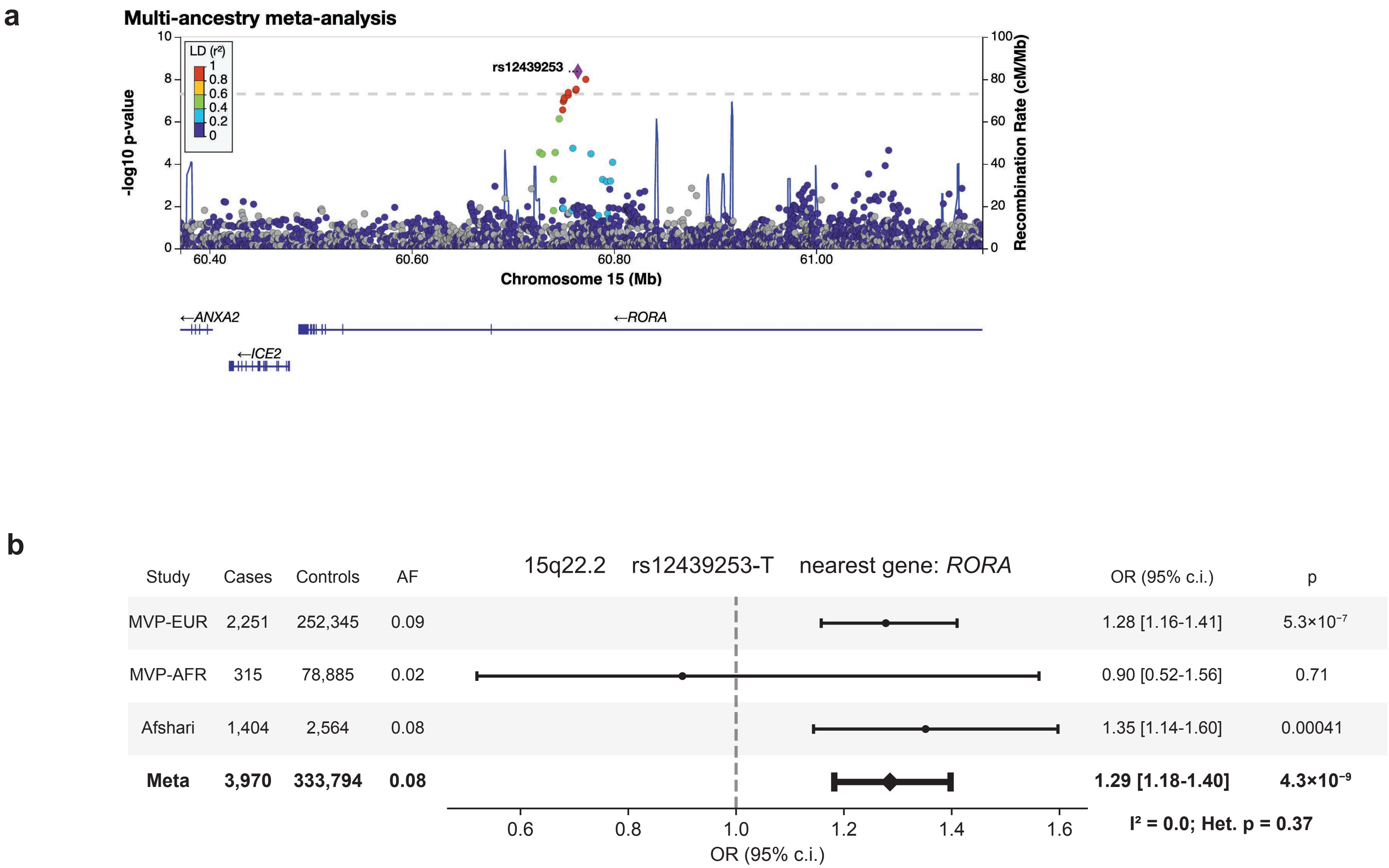

Supplementary Fig. 11. Regional Manhattan plot and forest plot for *RORA*. (a) LocusZoom regional Manhattan plot showing variant-level associations and local linkage disequilibrium (LD) structure of a significant multi-ancestry meta-analysis FECD genomic risk locus. Plots are presented in the order of their genomic coordinates. A European LD reference panel (1000 genomes) was used to color points in all plots. **(b)** Odds ratios (OR) and 95% confidence intervals for FECD at lead SNPs in Million Veteran Program European (EUR), admixed African (AFR), and Afshari[1] cohorts, and combined multi-ancestry meta-analysis (Meta). OR is not shown for six of twelve AFR index variants which did not meet the meta-analysis minor allele frequency cutoff of ≥1%; for these variants, “Meta” refers to the EUR-only meta-analysis.

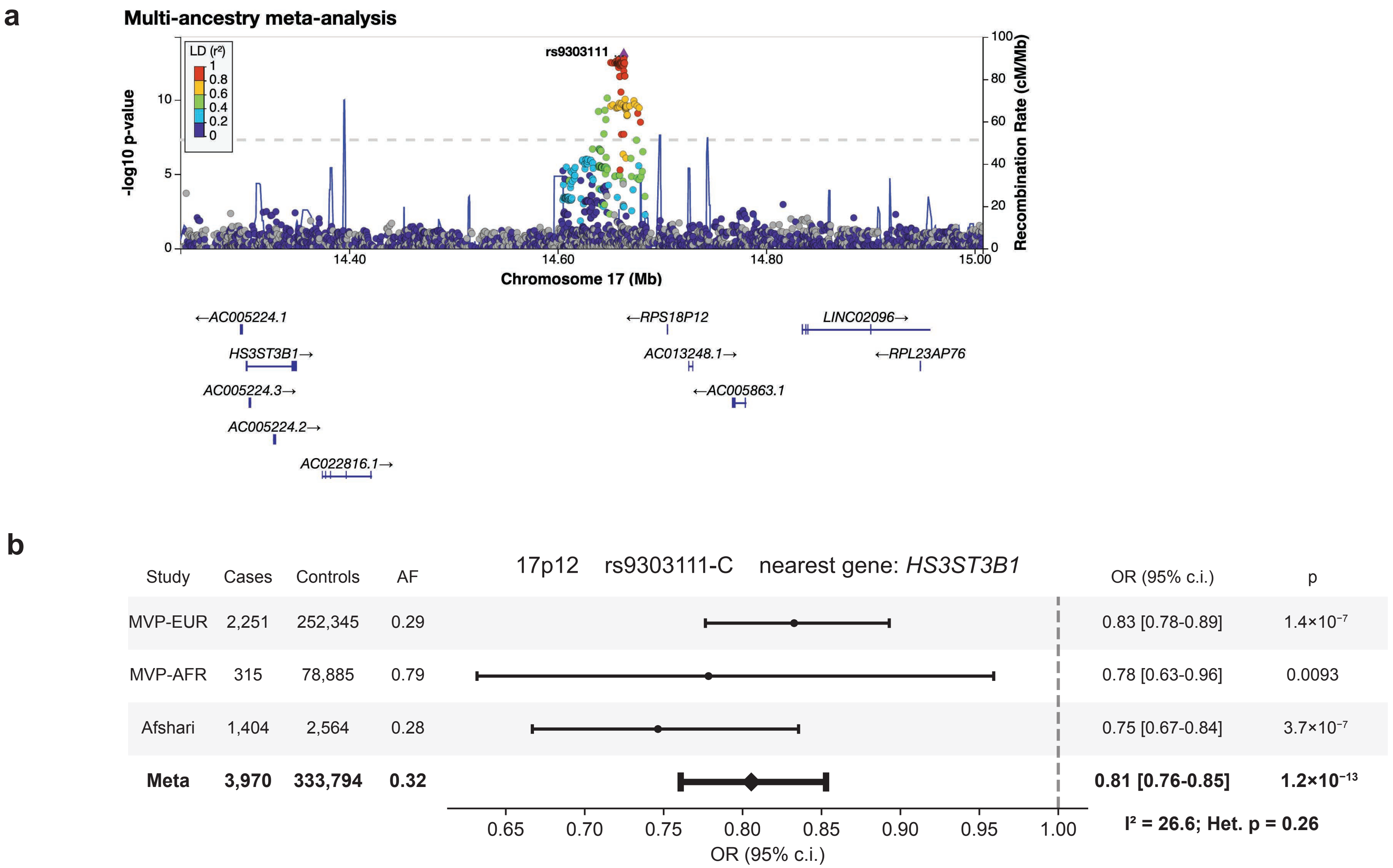

Supplementary Fig. 12. Regional Manhattan plot and forest plot for *HS3ST3B1*. (a) LocusZoom regional Manhattan plot showing variant-level associations and local linkage disequilibrium (LD) structure of a significant multi-ancestry meta-analysis FECD genomic risk locus. Plots are presented in the order of their genomic coordinates. A European LD reference panel (1000 genomes) was used to color points in all plots. **(b)** Odds ratios (OR) and 95% confidence intervals for FECD at lead SNPs in Million Veteran Program European (EUR), admixed African (AFR), and Afshari[1] cohorts, and combined multi-ancestry meta-analysis (Meta). OR is not shown for six of twelve AFR index variants which did not meet the meta-analysis minor allele frequency cutoff of ≥1%; for these variants, “Meta” refers to the EUR-only meta-analysis.

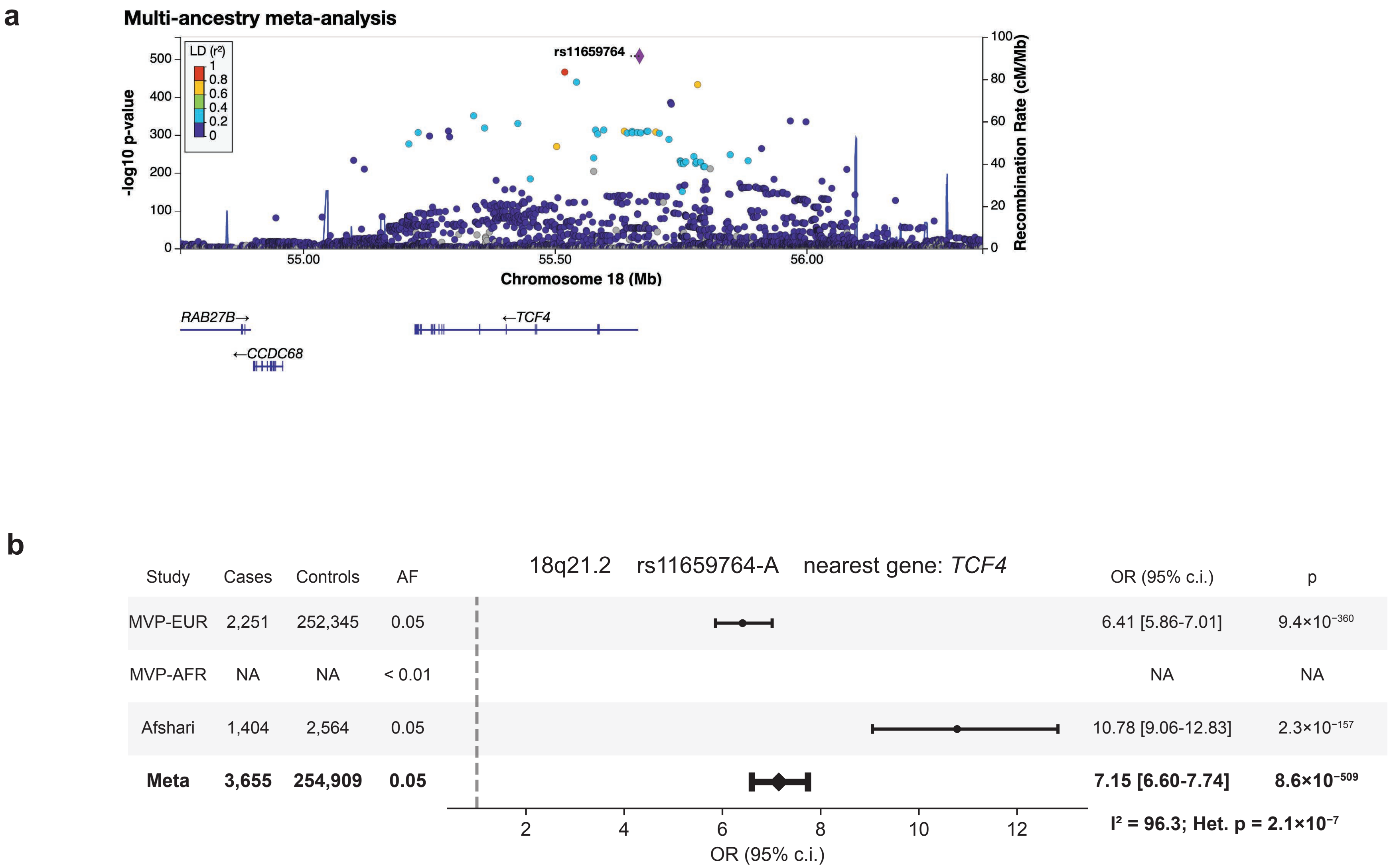

Supplementary Fig. 13. Regional Manhattan plot and forest plot for *TCF4*. (a) LocusZoom regional Manhattan plot showing variant-level associations and local linkage disequilibrium (LD) structure of a significant multi-ancestry meta-analysis FECD genomic risk locus. Plots are presented in the order of their genomic coordinates. A European LD reference panel (1000 genomes) was used to color points in all plots. **(b)** Odds ratios (OR) and 95% confidence intervals for FECD at lead SNPs in Million Veteran Program European (EUR), admixed African (AFR), and Afshari[1] cohorts, and combined multi-ancestry meta-analysis (Meta). OR is not shown for six of twelve AFR index variants which did not meet the meta-analysis minor allele frequency cutoff of ≥1%; for these variants, "Meta" refers to the EUR-only meta-analysis.

a

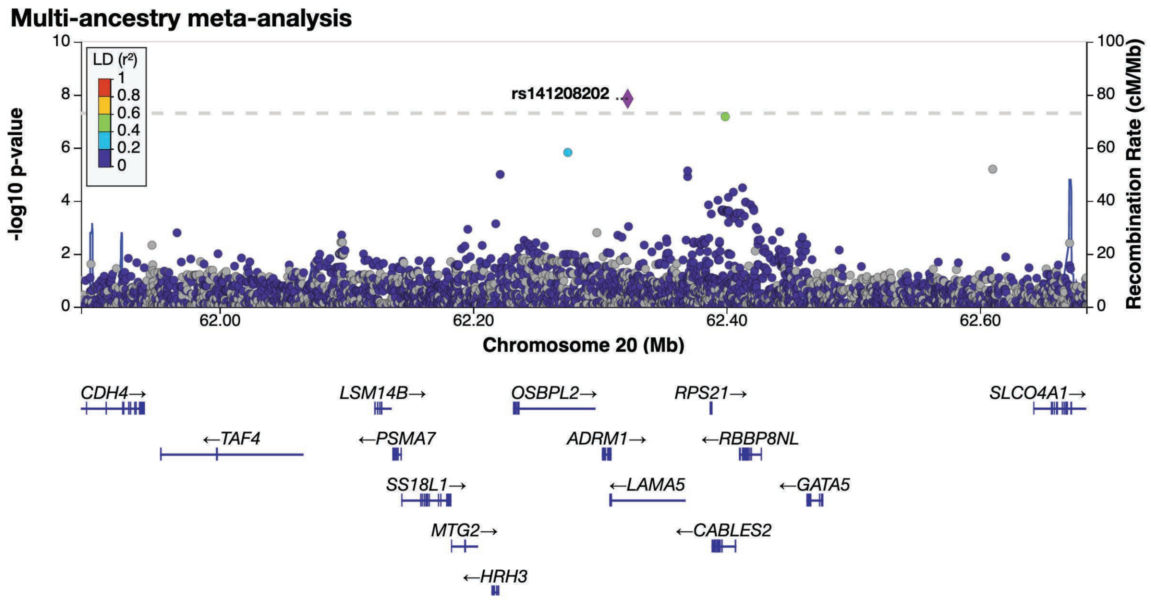

b

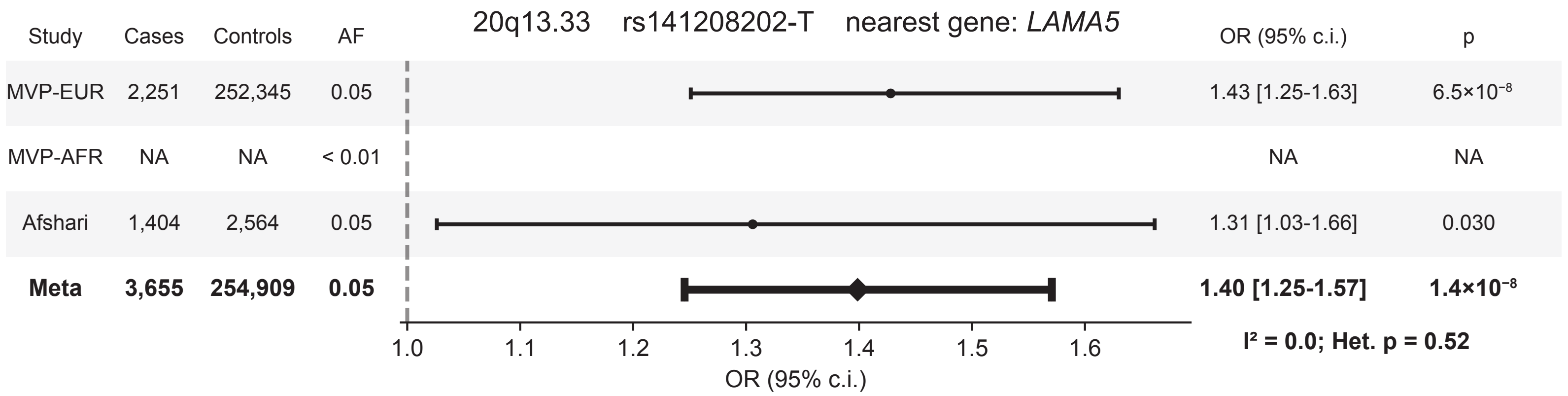

Supplementary Fig. 14. Regional Manhattan plot and forest plot for *LAMA5*. (a) LocusZoom regional Manhattan plot showing variant-level associations and local linkage disequilibrium (LD) structure of a significant multi-ancestry meta-analysis FECD genomic risk locus. Plots are presented in the order of their genomic coordinates. A European LD reference panel (1000 genomes) was used to color points in all plots. **(b)** Odds ratios (OR) and 95% confidence intervals for FECD at lead SNPs in Million Veteran Program European (EUR), admixed African (AFR), and Afshari[1] cohorts, and combined multi-ancestry meta-analysis (Meta). OR is not shown for six of twelve AFR index variants which did not meet the meta-analysis minor allele frequency cutoff of ≥1%; for these variants, "Meta" refers to the EUR-only meta-analysis.

a

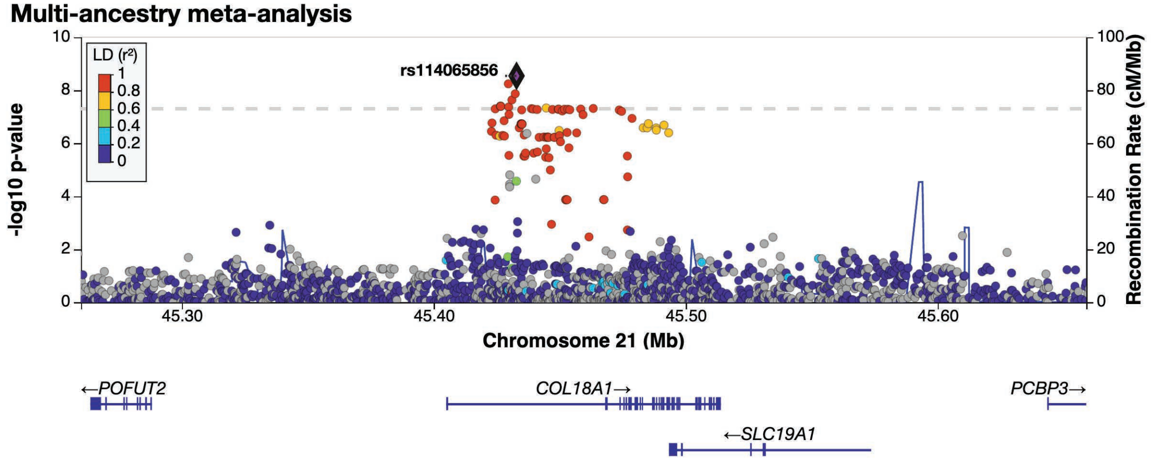

b

21q22.3 rs114065856-T nearest gene: *COL18A1*

Study	Cases	Controls	AF	OR (95% c.i.)	p
MVP-EUR	2,251	252,345	0.03	0.63 [0.51-0.78]	7.5×10^{-6}
MVP-AFR	315	78,885	0.05	0.77 [0.52-1.12]	0.17
Afshari	1,404	2,564	0.03	0.48 [0.35-0.68]	2.3×10^{-5}
**Meta**	**3,970**	**333,794**	**0.04**	**0.61 [0.52-0.72]**	$\mathbf{2.9\times10^{-9}}$

I^2 = 39.2; Het. p = 0.19

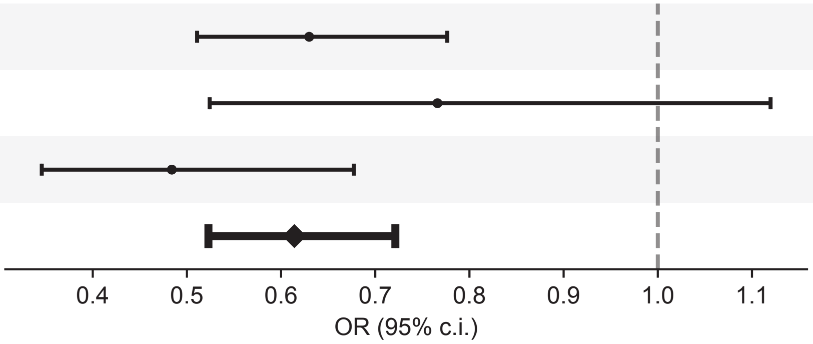

Supplementary Fig. 15. Regional Manhattan plot and forest plot for *COL18A1*. (a) LocusZoom regional Manhattan plot showing variant-level associations and local linkage disequilibrium (LD) structure of a significant multi-ancestry meta-analysis FECD genomic risk locus. Plots are presented in the order of their genomic coordinates. A European LD reference panel (1000 genomes) was used to color points in all plots. **(b)** Odds ratios (OR) and 95% confidence intervals for FECD at lead SNPs in Million Veteran Program European (EUR), admixed African (AFR), and Afshari[1] cohorts, and combined multi-ancestry meta-analysis (Meta). OR is not shown for six of twelve AFR index variants which did not meet the meta-analysis minor allele frequency cutoff of ≥1%; for these variants, "Meta" refers to the EUR-only meta-analysis.

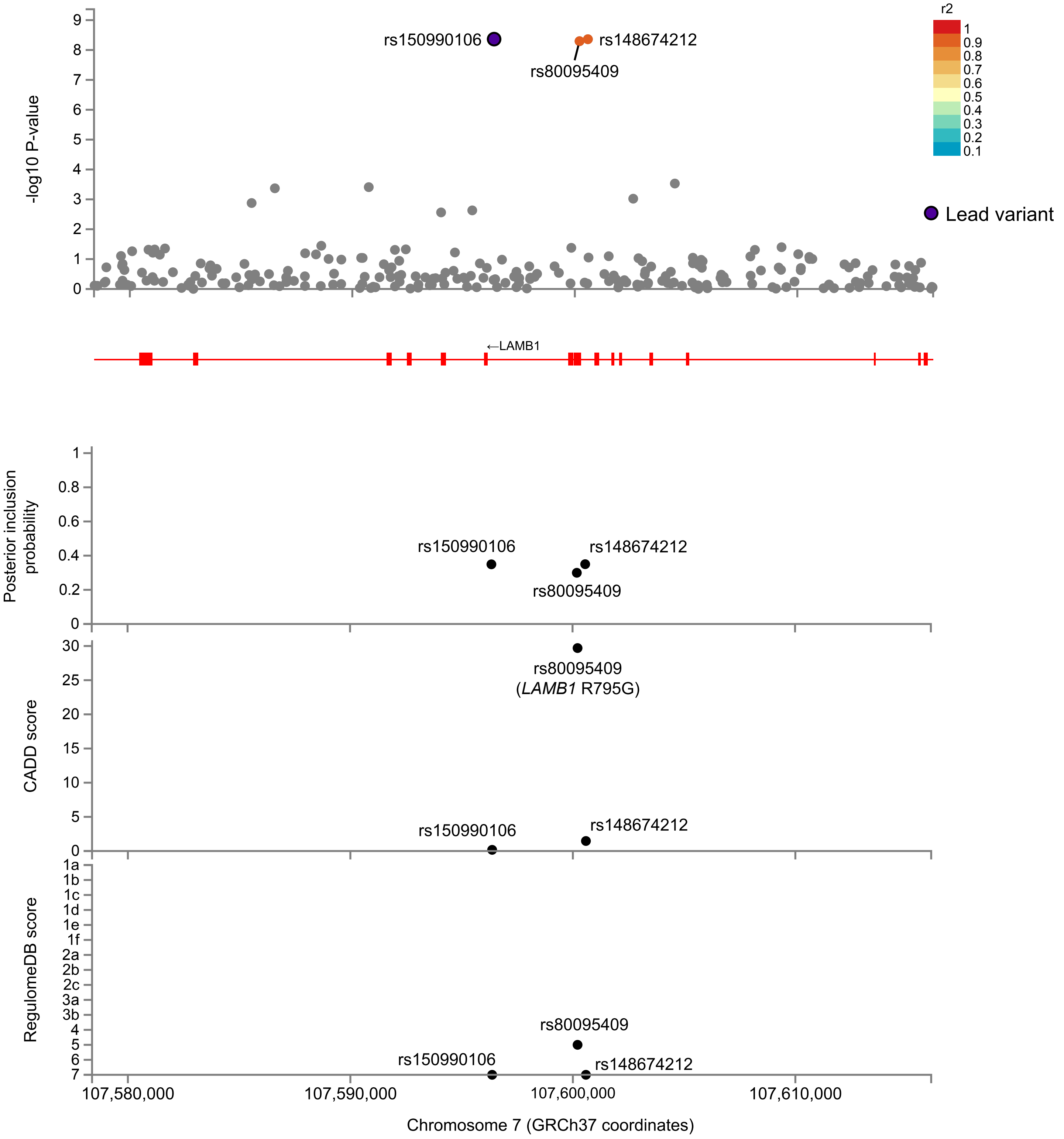

Supplementary Fig. 16. Novel causal variant prediction at *LAMB1*. Local Manhattan plots, finemapping posterior inclusion probabilities, Combined Annotation Dependent Depletion (CADD) scores, and RegulomeDB scores for significant Fuchs endothelial corneal dystrophy associations in the European meta-analysis.

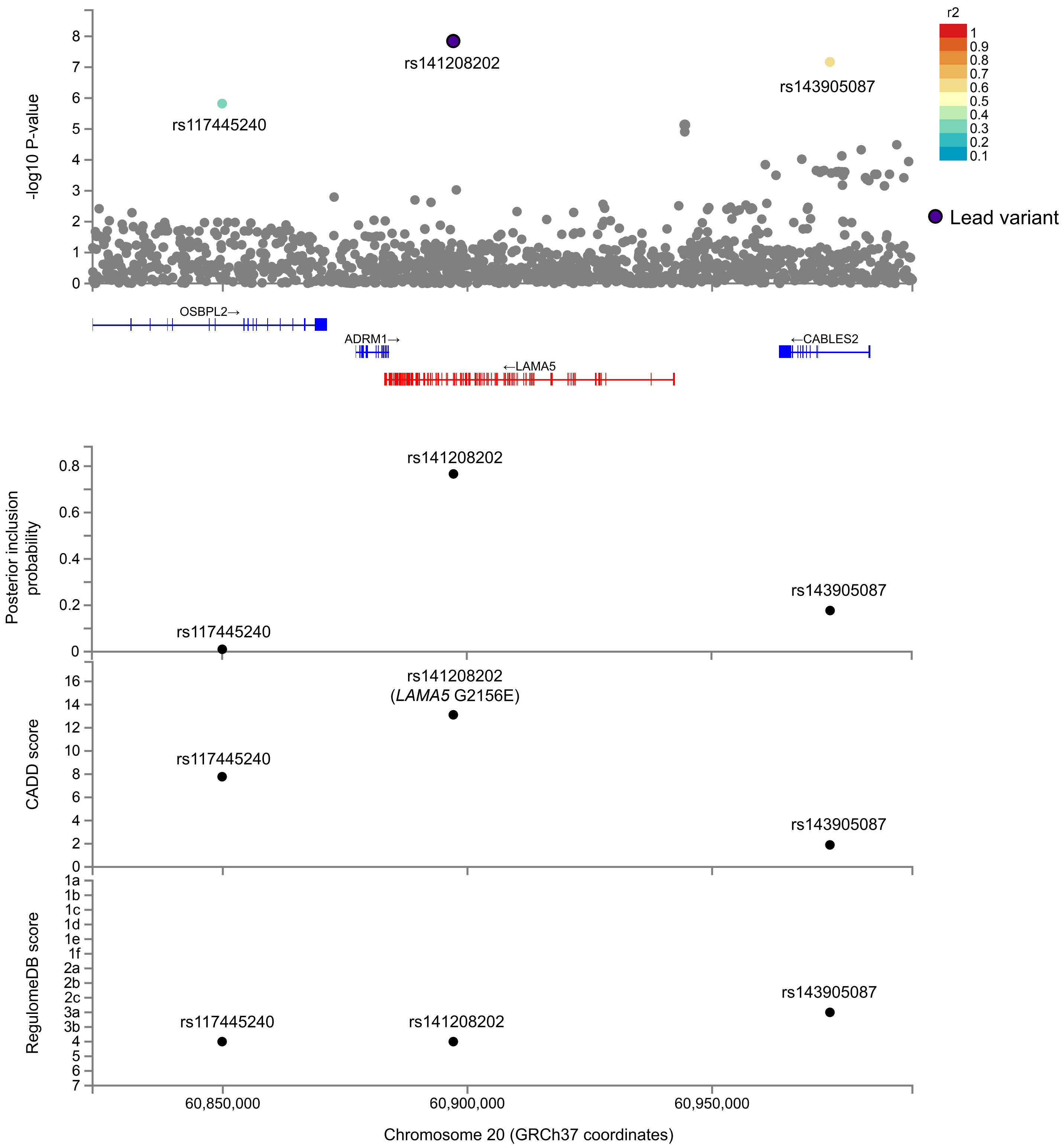

Supplementary Fig. 17. Novel causal variant prediction at *LAMA5*. Local Manhattan plots, finemapping posterior inclusion probabilities, Combined Annotation Dependent Depletion (CADD) scores, and RegulomeDB scores for significant Fuchs endothelial corneal dystrophy associations in the European meta-analysis.

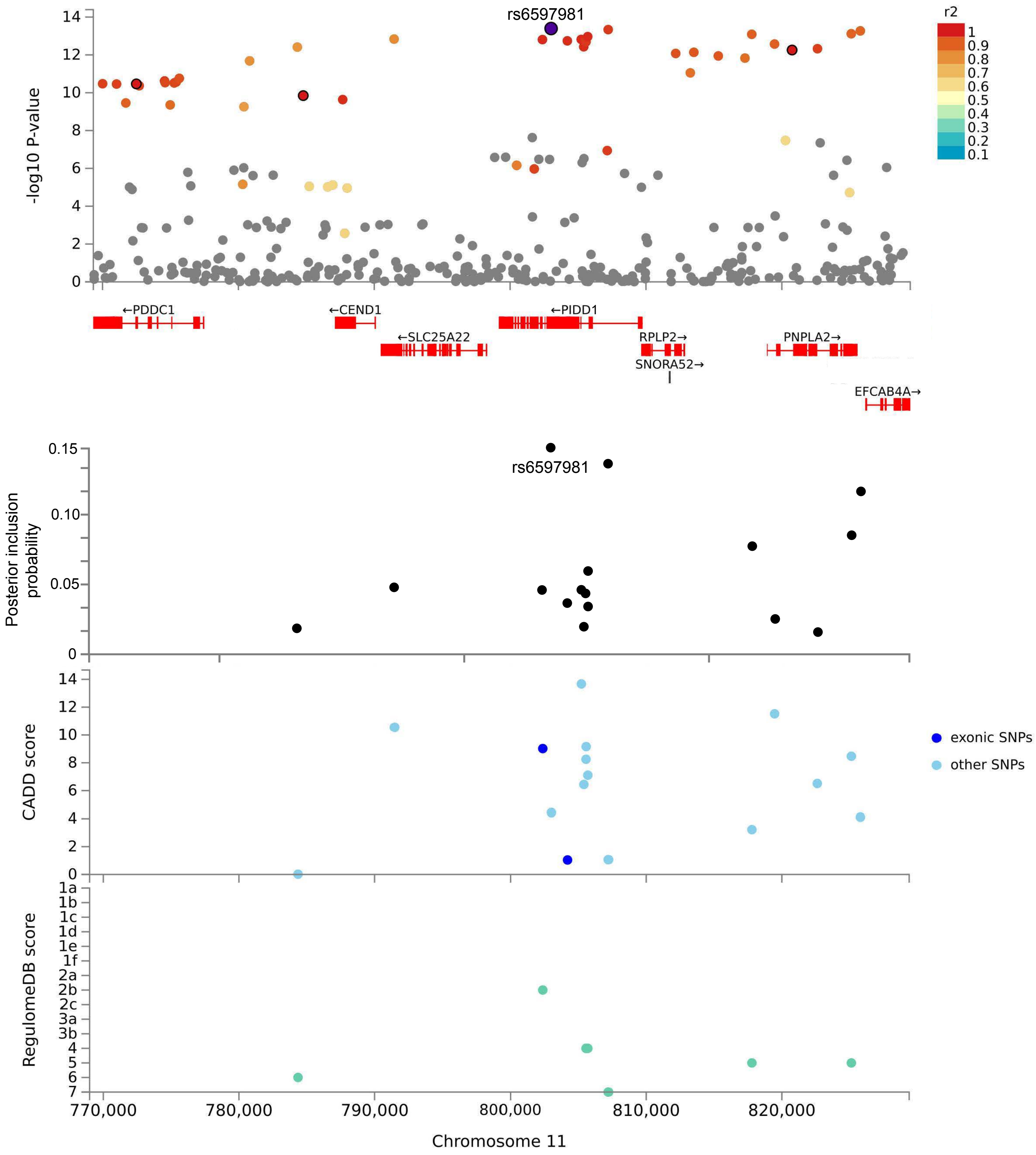

Supplementary Fig. 18. Novel causal variant prediction at *PIDD1*. Local Manhattan plots, finemapping posterior inclusion probabilities, Combined Annotation Dependent Depletion (CADD) scores, and RegulomeDB scores for significant Fuchs endothelial corneal dystrophy associations in the European meta-analysis.

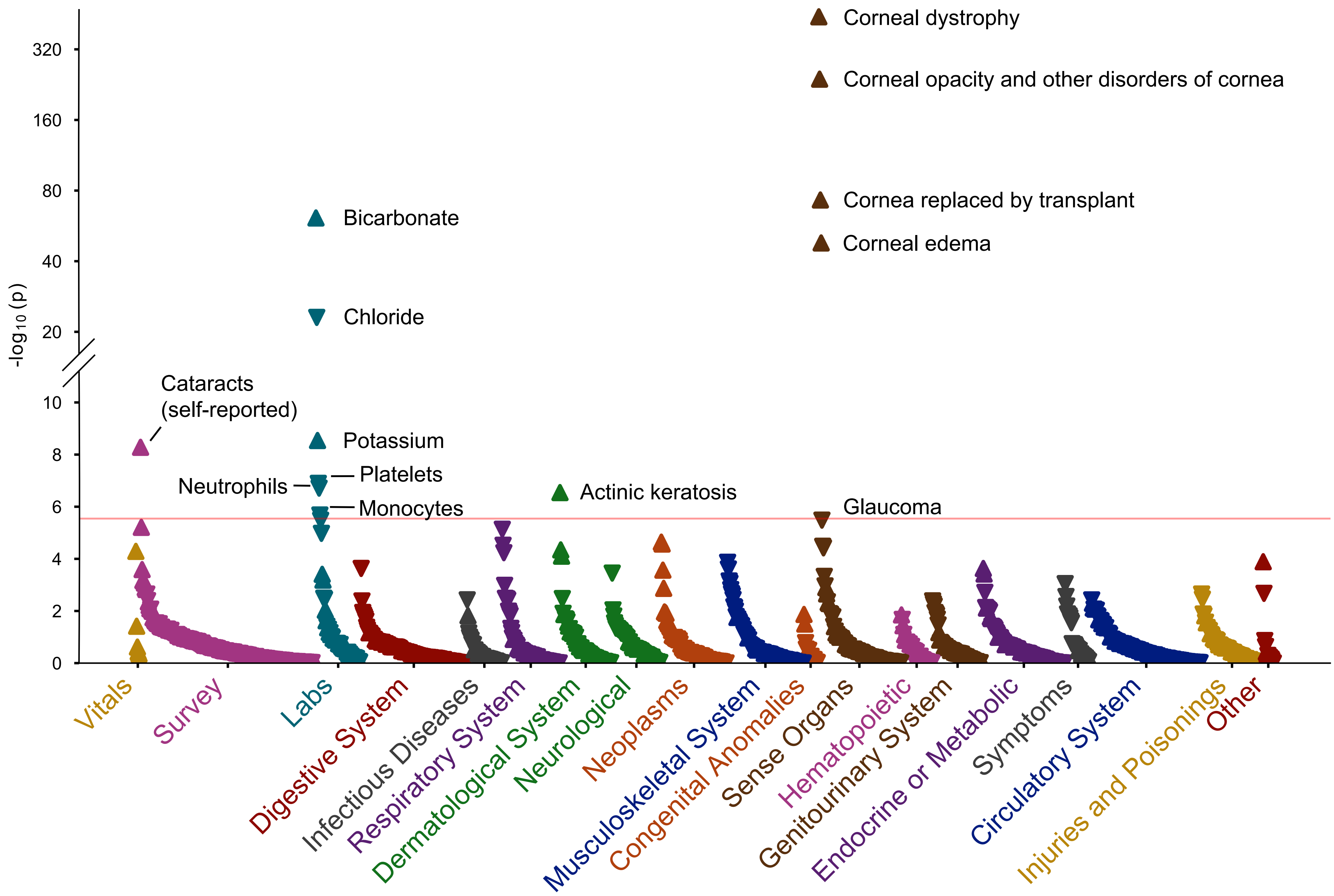

Supplementary Fig. 19. rs11659764 variant-level PheWAS. Phenome-wide associations with rs11659764, the lead Fuchs endothelial corneal dystrophy (FECD) variant at *TCF4*. Phenotypes are grouped by category those reaching multiple testing corrected significance ($P<2.9\times10^{-6}$; red line) are labeled. Point direction indicates positive or negative effect. Y-axis break indicates switch to logarithmic scale. FECD (not shown) had the most significant association ($P=5.01\times10^{-658}$). All lab measures were rank inverse normal transformed.

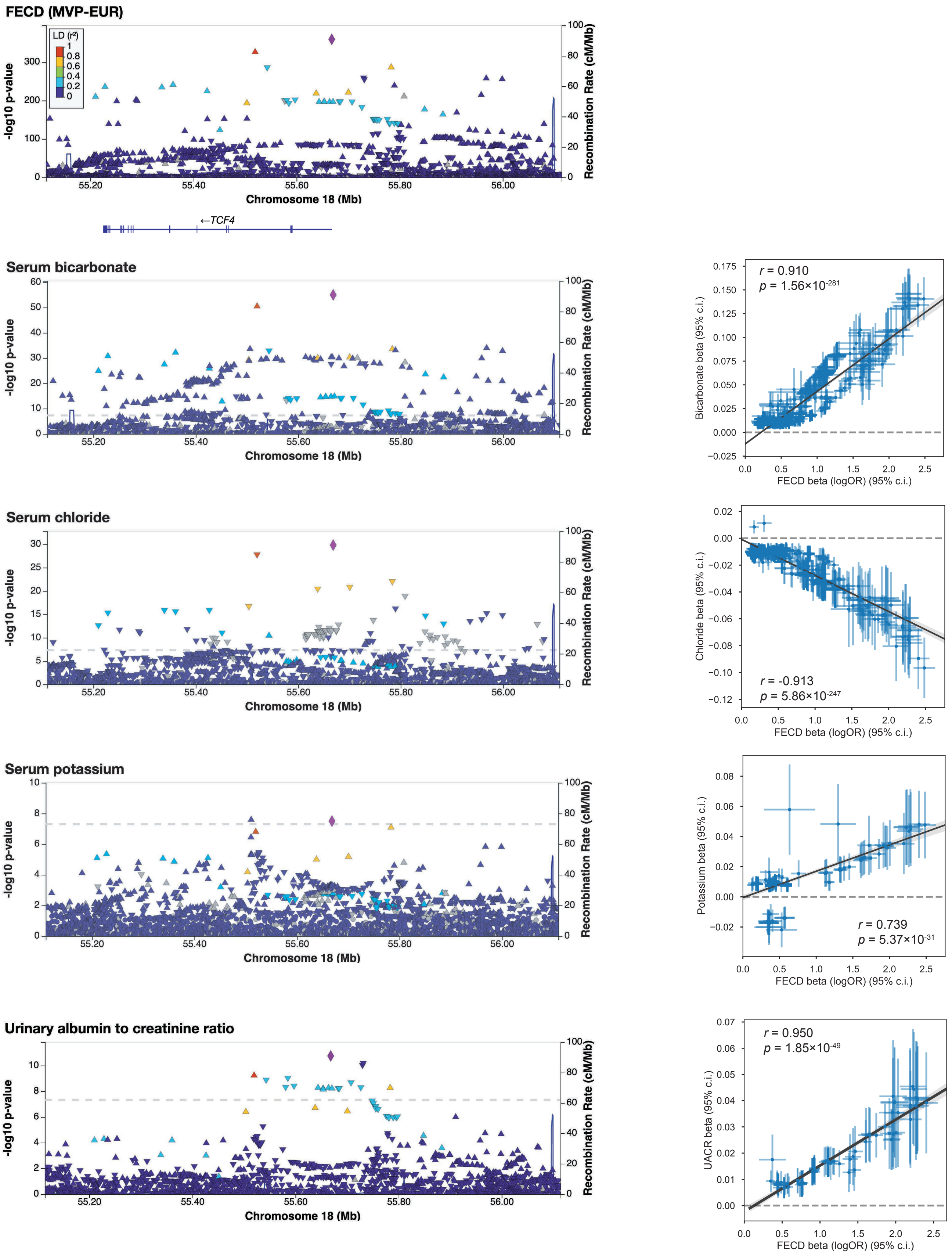

Supplementary Fig. 20. Colocalization with renal traits at *TCF4*. Left: For Million Veteran Program EUR participants, regional Manhattan plots at the *TCF4* locus with FECD, serum bicarbonate, serum chloride, serum potassium, and urinary albumin-to-creatinine ratio. **Right:** Effects comparison scatterplots for variants in the *TCF4* locus (chr18:54,500,000 to 56,500,000; only those points with $P<0.001$) and 95% confidence interval (c.i.). FECD effects are shown as logarithm of odds ratios. Trendline, correlation coefficients (r) and associated P-values are shown for scatterplots.

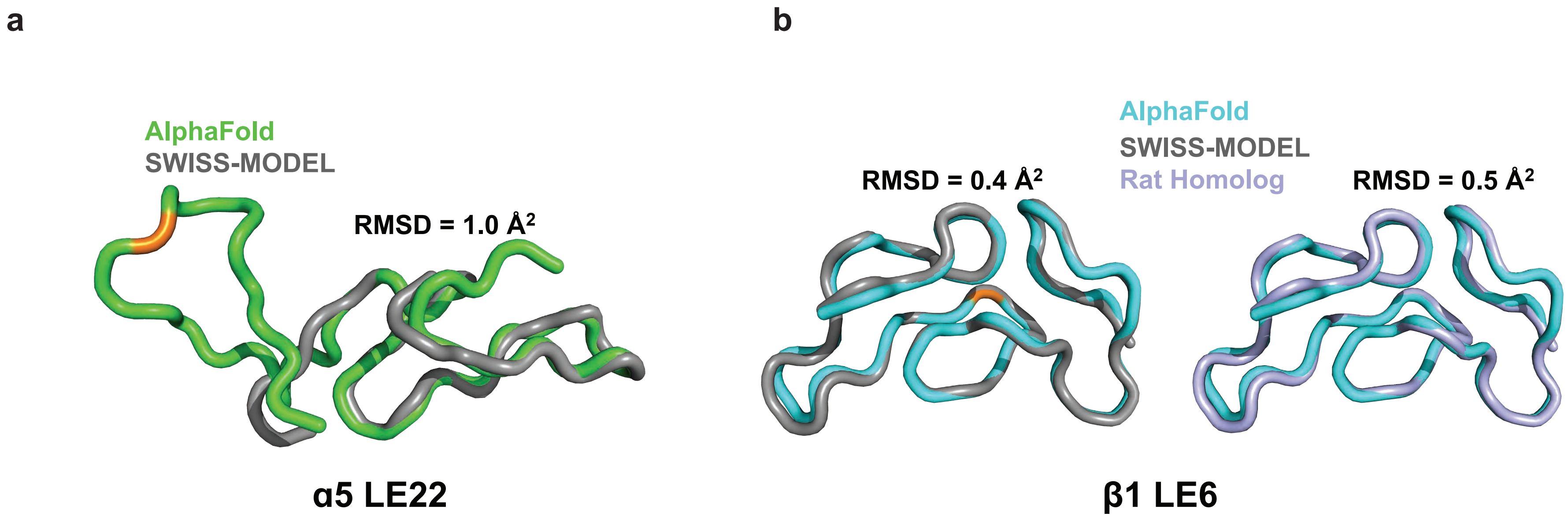

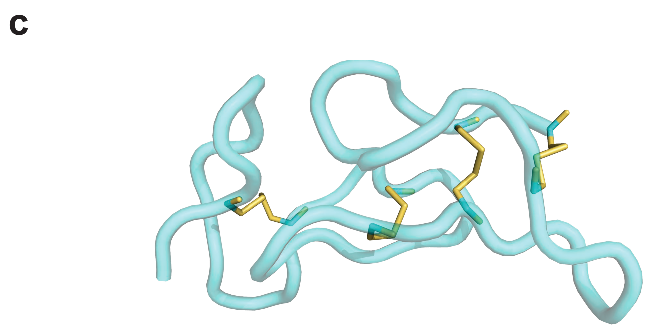

d

Mutation	H-BOND(count)	Predicted G (kcal/mol)
α5-LE22 G2156E	0	-0.266
β1 LE6 R795G	-3	-1.226

Supplementary Fig. 21. Predicted impact of mutation on LE domain structure. Missense mutations associated with risk of Fuchs endothelial corneal dystrophy in **(a)** and **(b)** are colored orange. **(a)** The homolog template for α5 does not cover the entire target sequence, hence the predicted homology model does not include a portion of the domain. However, given the excellent agreement between the AlphaFold 2 (AF2) prediction and the homology model for the existing portion of the domain of α5 LE22, it is likely that AF2 is providing a feasible conformation of the α5 LE22 loop missing in the template structure. **(b)** The AF2 prediction for β1 LE6 has excellent agreement with the crystallized rat homolog (67% sequence identity) as well as the SWISS-MODEL homology model. **(c)** AF2 prediction of β1 LE6 with four disulfide links that fortify the tertiary structure of the domain displayed as yellow sticks. **(d)** Change in hydrogen bond content and Gibbs free energy introduced by the mutations. The substitution of a glycine with glutamic acid in A5 LE22 does not result in loss or gain of hydrogen bonds within the domain. Meanwhile, the replacement of the arginine by a glycine in β1 LE6 results in a loss of 3 hydrogen bonds.

Supplementary References

1. Afshari, N. A. *et al.* Genome-wide association study identifies three novel loci in Fuchs endothelial corneal dystrophy. *Nat. Commun.* **8**, 14898 (2017).
