# Supplementary material for: A multi-ancestry GWAS of Fuchs corneal dystrophy highlights the contributions of laminins, collagen, and endothelial cell regulation
Source: Commun Biol. 2024 Apr 6;7:418. doi: 10.1038/s42003-024-06046-3 (PMC10998918; doi:10.1038/s42003-024-06046-3)
Supplement: Supplementary file 3 — Description of Additional Supplementary Files [file 42003_2024_6046_MOESM3_ESM.pdf]

Description of Additional Supplementary Files

File name: Supplementary Data 1
Description: Study overview.

File name: Supplementary Data 2
Description: Significant MVP discovery loci.

File name: Supplementary Data 3
Description: Genome-wide significant loci in the multi-ancestry meta-analysis of Fuchs Endothelial Corneal Dystrophy (FECD), by individual cohort.

File name: Supplementary Data 4
Description: Ocular trait pleiotropy.

File name: Supplementary Data 5
Description: Comparison of corneal effect sizes.

File name: Supplementary Data 6
Description: The source data behind Fig. 3 in the paper.

File name: Supplementary Data 7
Description: Association of FECD status with polygenic scores (PGS).

File name: Supplementary Data 8
Description: Index variant phenome-wide association scans (PheWAS).

File name: Supplementary Data 9
Description: Colocalization results.
