# Supplementary material for: A multi-ancestry GWAS of Fuchs corneal dystrophy highlights the contributions of laminins, collagen, and endothelial cell regulation
Source: Commun Biol. 2024 Apr 6;7:418. doi: 10.1038/s42003-024-06046-3 (PMC10998918; doi:10.1038/s42003-024-06046-3)
Supplement: Supplementary file 5 — Reporting Summary [file 42003_2024_6046_MOESM5_ESM.pdf]

nature portfolio

Corresponding author(s): Iyengar, Sudha
Peachey, Neal

Last updated by author(s): Feb 12, 2024

Reporting Summary

Nature Portfolio wishes to improve the reproducibility of the work that we publish. This form provides structure for consistency and transparency in reporting. For further information on Nature Portfolio policies, see our Editorial Policies and the Editorial Policy Checklist.

Statistics

For all statistical analyses, confirm that the following items are present in the figure legend, table legend, main text, or Methods section.

n/a	Confirmed	
☐	☒	The exact sample size (*n*) for each experimental group/condition, given as a discrete number and unit of measurement
☒	☐	A statement on whether measurements were taken from distinct samples or whether the same sample was measured repeatedly
☐	☒	The statistical test(s) used AND whether they are one- or two-sided<br>*Only common tests should be described solely by name; describe more complex techniques in the Methods section.*
☐	☒	A description of all covariates tested
☐	☒	A description of any assumptions or corrections, such as tests of normality and adjustment for multiple comparisons
☐	☒	A full description of the statistical parameters including central tendency (e.g. means) or other basic estimates (e.g. regression coefficient) AND variation (e.g. standard deviation) or associated estimates of uncertainty (e.g. confidence intervals)
☐	☒	For null hypothesis testing, the test statistic (e.g. *F*, *t*, *r*) with confidence intervals, effect sizes, degrees of freedom and *P* value noted<br>*Give P values as exact values whenever suitable.*
☒	☐	For Bayesian analysis, information on the choice of priors and Markov chain Monte Carlo settings
☒	☐	For hierarchical and complex designs, identification of the appropriate level for tests and full reporting of outcomes
☐	☒	Estimates of effect sizes (e.g. Cohen's *d*, Pearson's *r*), indicating how they were calculated

Our web collection on statistics for biologists contains articles on many of the points above.

Software and code

Policy information about availability of computer code

Data collection	Data collection: Clinical and genetic data in MVP were collected as previously described in PMID 37170406 and 26441289.
Data analysis	HARE (https://github.com/tanglab/HARE); SHAPEIT4; Minimac4; SAIGE (v1.1.6.2); bcftools v1.16; GCTA-mtCOJO (v1.93.2 beta); R (v4.0.2); LDSC (v1.0.1); MungeSumstats (R; v1.7.8); Functional Mapping and Annotation of Genome-Wide Association Studies (FUMA) web server (v1.4.2); Coloc (R; v5.2.1); AlphaFold 2 (v2.2.4); SWISS-MODEL; DUET (v0.5)

For manuscripts utilizing custom algorithms or software that are central to the research but not yet described in published literature, software must be made available to editors and reviewers. We strongly encourage code deposition in a community repository (e.g. GitHub). See the Nature Portfolio guidelines for submitting code & software for further information.

Data

Policy information about availability of data

All manuscripts must include a data availability statement. This statement should provide the following information, where applicable:

- Accession codes, unique identifiers, or web links for publicly available datasets
- A description of any restrictions on data availability
- For clinical datasets or third party data, please ensure that the statement adheres to our policy

The full summary level association data from the meta-analysis and individual population association analyses in MVP will be available via the dbGaP study accession number phs001672.

Research involving human participants, their data, or biological material

Policy information about studies with human participants or human data. See also policy information about sex, gender (identity/presentation), and sexual orientation and race, ethnicity and racism.

Reporting on sex and gender	All analyses were performed using sex assessed using genetic markers, and the term "sex" is used throughout the manuscript.
Reporting on race, ethnicity, or other socially relevant groupings	Analyses were performed using HARE-defined harmonized race/ethnicity, described in PMID 31564439 (ref 70), and this characteristic is referred to as "ancestry" throughout the manuscript.
Population characteristics	Characteristics of the MVP cohort and Afshari et al. replication cohort are provided in PMID 37170406 and 28358029, respectively.
Recruitment	Subjects in MVP were recruited from a population of United States Veterans receiving care within the Veterans Health Administration (VHA). More details on recruitment are previously provided in PMID 37170406. As of 2022, Million Veteran Program participants represent approximately 10% of the total Veteran population enrolled in the VHA.
Ethics oversight	The VA Office of Research and Development central IRB approved the MVP024 study protocol.

Note that full information on the approval of the study protocol must also be provided in the manuscript.

Field-specific reporting

Please select the one below that is the best fit for your research. If you are not sure, read the appropriate sections before making your selection.

☒ Life sciences ☐ Behavioural & social sciences ☐ Ecological, evolutionary & environmental sciences

For a reference copy of the document with all sections, see nature.com/documents/nr-reporting-summary-flat.pdf

Life sciences study design

All studies must disclose on these points even when the disclosure is negative.

Sample size	Sample size was determined from summing the case and control numbers in each component study in the meta-analysis.
Data exclusions	Participant exclusions were made using standard criteria for GWAS, which included withdrawn consent, data missingness, and poor quality genotyping.
Replication	Replication for FECD was performed in the Afshari et al. cohort (PMID 28358029).
Randomization	Randomization was not applicable to this study.
Blinding	Blinding was not applicable to this study.

Reporting for specific materials, systems and methods

We require information from authors about some types of materials, experimental systems and methods used in many studies. Here, indicate whether each material, system or method listed is relevant to your study. If you are not sure if a list item applies to your research, read the appropriate section before selecting a response.

Materials & experimental systems

n/a	Involved in the study	
☒	☐	Antibodies
☒	☐	Eukaryotic cell lines
☒	☐	Palaeontology and archaeology
☒	☐	Animals and other organisms
☒	☐	Clinical data
☒	☐	Dual use research of concern
☒	☐	Plants

Methods

n/a	Involved in the study	
☒	☐	ChIP-seq
☒	☐	Flow cytometry
☒	☐	MRI-based neuroimaging
